# Microbial Community Structure and Functional Potential in Cultivated and Native Tallgrass Prairie Soils of the Midwestern United States

**DOI:** 10.3389/fmicb.2018.01775

**Published:** 2018-08-15

**Authors:** Rachel Mackelprang, Alyssa M. Grube, Regina Lamendella, Ederson da C. Jesus, Alex Copeland, Chao Liang, Randall D. Jackson, Charles W. Rice, Stefanie Kapucija, Bayan Parsa, Susannah G. Tringe, James M. Tiedje, Janet K. Jansson

**Affiliations:** ^1^Department of Biology, California State University, Northridge, Northridge, CA, United States; ^2^Department of Biology, Juniata College, Huntingdon, PA, United States; ^3^Center for Microbial Ecology, Michigan State University, East Lansing, MI, United States; ^4^Great Lakes Bioenergy Research Center, U.S. Department of Energy, University of Wisconsin–Madison, Madison, WI, United States; ^5^U.S. Department of Energy Joint Genome Institute, Walnut Creek, CA, United States; ^6^Institute of Applied Ecology, Chinese Academy of Sciences, Shenyang, China; ^7^Department of Agronomy, University of Wisconsin–Madison, Madison, WI, United States; ^8^Department of Agronomy, Kansas State University, Manhattan, KS, United States; ^9^Earth and Biological Sciences Directorate, Pacific Northwest National Laboratory, Richland, WA, United States

**Keywords:** soil microbiome, land management, metagenomics, native prairie, climate change, carbon cycle, nitrogen cycle

## Abstract

The North American prairie covered about 3.6 million-km^2^ of the continent prior to European contact. Only 1–2% of the original prairie remains, but the soils that developed under these prairies are some of the most productive and fertile in the world, containing over 35% of the soil carbon in the continental United States. Cultivation may alter microbial diversity and composition, influencing the metabolism of carbon, nitrogen, and other elements. Here, we explored the structure and functional potential of the soil microbiome in paired cultivated-corn (at the time of sampling) and never-cultivated native prairie soils across a three-states transect (Wisconsin, Iowa, and Kansas) using metagenomic and 16S rRNA gene sequencing and lipid analysis. At the Wisconsin site, we also sampled adjacent restored prairie and switchgrass plots. We found that agricultural practices drove differences in community composition and diversity across the transect. Microbial biomass in prairie samples was twice that of cultivated soils, but alpha diversity was higher with cultivation. Metagenome analyses revealed denitrification and starch degradation genes were abundant across all soils, as were core genes involved in response to osmotic stress, resource transport, and environmental sensing. Together, these data indicate that cultivation shifted the microbiome in consistent ways across different regions of the prairie, but also suggest that many functions are resilient to changes caused by land management practices – perhaps reflecting adaptations to conditions common to tallgrass prairie soils in the region (e.g., soil type, parent material, development under grasses, temperature and rainfall patterns, and annual freeze-thaw cycles). These findings are important for understanding the long-term consequences of land management practices to prairie soil microbial communities and their genetic potential to carry out key functions.

## Introduction

The original North American prairie was a 3.6 million-km^2^ expanse of fertile soil (Mollisols). This region is highly productive agriculturally and the majority of the original prairie has been cultivated (Samson and Knopf, 1994). Besides replacing a species-rich plant community with monoculture, land management induces changes in soil physicochemical characteristics. Of particular importance to global biogeochemical cycles is the impact of human activities on nitrogen and carbon storage. In 2016, agriculture was the source of 8.6% of total greenhouse gas emissions in the United States (USEPA, 2018). Thirty one to 39% of the total soil organic carbon (SOC) stocks of the conterminous United States are stored in prairie soils (Guo et al., 2006). Tillage, fertilization, intensive cropping, and erosion causes SOC losses of 20–60% (Stauffer et al., 1940; Mann, 1986; Brye et al., 2001, 2002; Kucharik et al., 2001; Guo and Gifford, 2002; Sanford G.R. et al., 2012; Sanderman et al., 2017). Fertilizer application and other agricultural management practices induce N_2_O production, making croplands responsible for 76.7% of United States N_2_O emissions into the atmosphere (USEPA, 2018).

Microbial communities drive carbon and nitrogen cycles in soils. Thus, understanding how agricultural practices impact microbial communities is critical for predicting future greenhouse gas emissions. However, our understanding of microbial diversity within prairie ecosystems and how prairie soil microbiomes contribute to cycling of carbon, nitrogen, and other nutrients is still developing. Disturbances related to agriculture, such as tilling, fertilization, irrigation, and burning change soil properties and alter microbial community structure and functional capacity (Vitousek et al., 1997; Bending et al., 2004; Mao et al., 2011; Orr et al., 2011; Habig and Swanepoel, 2015; Jesus et al., 2015; Oates et al., 2016; Zhang et al., 2017). Fertilization adds mineral N to soils, which is processed by microbial communities through nitrification and denitrification pathways, producing N_2_O (Firestone and Davidson, 1989). N amendment shifts microbial community composition and functional capacity through changes to taxonomic richness (Coolon et al., 2013), activity (Marx et al., 2001; Ramirez et al., 2012), biomass (Ramirez et al., 2012), increases in active copiotrophic taxa (Fierer et al., 2012), and community composition (Ramirez et al., 2012; Leff et al., 2015). Tillage changes soil physicochemical properties (Phillips et al., 1980; Ismail et al., 1994) and concomitantly alters microbial community structure. It increases the abundance of aerobes, facultative anaerobes, and denitrifiers in near-surface soils (Doran, 1980) and causes changes in biomass (Guo et al., 2006), diversity, and activity (Habig and Swanepoel, 2015; Mbuthia et al., 2015; Nivelle et al., 2016). Monoculture cropping, some pesticide applications, and organic management practices also alter soil microbial community structure and diversity (Bending et al., 2000, 2004; Figuerola et al., 2014; Duncan et al., 2016; Liang et al., 2016; Pose-Juan et al., 2017; Zhang et al., 2017; Zhao et al., 2018).

Broad-scale comparisons between geographic locations, crop types, native prairie, and restored prairie ecosystems capture differences in community structure and function driven by the aggregate effects of cropping. Soil depth, crop systems (crop species, monoculture, and annual versus perennial), and soil variables all contribute to community assemblages and biomass (Acosta-Martinez et al., 2008; Habig and Swanepoel, 2015; Liang et al., 2016; Oates et al., 2016; Zhang et al., 2017). Community composition and function in grassland soils have also been found to vary across the world, likely due to differences in soil pH, climate, and plant communities (Fierer et al., 2013; Leff et al., 2015). Jesus et al. (2015) found that soil type and geographic distance drove community structure in recently established plots but that plant species became a dominant driver over long-term cultivation.

In the interest of restoring native habitat, mitigating biodiversity loss, preserving soil integrity, and investigating sustainable agriculture, grassland restoration is becoming more common in prairie ecosystems (Jangid et al., 2009; Barber et al., 2017). The magnitude and timing of the restoration of microbial community structure remains unclear. Some studies suggest that community response is rapid, occurring in less than a decade after restoration (Herzberger et al., 2014; Duncan et al., 2016; Barber et al., 2017). Other studies suggest that microbial community restoration is a long-term process occurring on the order of decades (Jangid et al., 2009).

Our understanding of microbial diversity within prairie ecosystems and how prairie soil microbial communities contribute to cycling of nutrients is still developing. Here we aimed to gain a better understanding of the effects of land management, specifically long-term cultivation, on soil microbial communities and their potential to carry out key soil processes in the region of the United States that had previously been dominated by prairie. We performed 16S rRNA gene sequencing, lipid analysis, and deep shotgun metagenomic sequencing in cultivated and native prairie soils across a three state transect. We asked how geographic location and cultivation practices influenced microbial community composition and about the capacity of soil microbes to cycle carbon, nitrogen, and other nutrients. An important part of our design was to compare long-term (>50 years) cultivated and never-cultivated sites that were otherwise matched (paired) with respect to soil and landform characteristics. Results from this study serve as a baseline for understanding the impacts of land management on soil communities, and consequently facilitate functional predictions of the impacts of cultivation on carbon and nutrient cycling processes.

## Materials and Methods

### Sampling Sites

Three native tallgrass prairie sites representative of the U.S. Midwest prairie ecosystem were studied: Manhattan, Kansas (KS); Morris Prairie, Iowa (IA); and Goose Pond Prairie, Wisconsin (WI). These sites constitute a southwest to northeast transect across what was originally tallgrass prairie, but is now mostly converted to highly productive annual crop agriculture. At each location, a nearby long-term agricultural site was selected that matched the never-tilled (remnant) prairie site in soil type, texture, slope, aspect, and drainage. A switchgrass and restored prairie plot adjacent to the corn plot were also included at the Wisconsin site. In the case of non-switchgrass cultivated sites, samples were taken when sites were planted to corn (a complete description of the sites and their history is in **Supplementary Methods**). All sites were sampled in 2009 during active plant growth and optimum soil moisture conditions: Wisconsin-June 24, Iowa-June 26, and Kansas-August 7. Seven samples were taken at each of the sites with a 1-cm diameter soil corer to a depth of 12 cm. A reference sample (defined as 0 m) and six additional cores were sampled in two directions from the reference (90 degree angle) at 1 cm, 1 m, and 10 m (**Supplementary Figure S1**). The corn sites were sampled between the rows. At this plant stage few roots were sampled. The litter layer was removed and the soil core extruded into a plastic bag. The eighth sample was a larger volume (500 g) sample taken adjacent to the reference core (the apex) designed for soil chemical and physical analyses. All samples were immediately placed on ice, stored locally under refrigeration, and shipped overnight on blue ice and kept cold until DNA was extracted or frozen until the soil chemistry analyzed. A subsample of approximately 3.2 or 6.4 g (if sufficient DNA was not obtained from the 3.2 g sample) from the reference core (0 m) was used for metagenomic sequencing. Subsamples from all eight cores were used for 16S rRNA gene sequencing and for lipid analysis.

### Soil Characterization

All soil chemical and physical attributes were analyzed at the Michigan State University Soil and Plant Nutrient Laboratory except for the boron, sulfur, and aluminum analyses, which were done by A&L Great Lakes Laboratories using the Mehlich 3 method. The chemical analyses were those validated for reflecting bioavailable elements in soils of the North Central region of the United States (46) plus the chemical specific methods of Bradstreet (1965), Huffman and Barbarick (2008), and the United States Environmental Protection Agency (USEPA, 1993). Physical (texture) analysis was by the hydrometer method of Bouyoucos (1951) (see **Supplementary Table S1**).

### DNA Extraction and 16S rRNA Gene Sequencing

DNA was extracted from 250 mg soil portions using the PowerSoil^®^ DNA isolation kit (Mo Bio Laboratories, Carlsbad, CA, United States) according to the manufacturer’s protocol. Multiple extractions were performed for each homogenized soil sample to obtain approximately 10 μg/sample. The V6–V8 region of the small subunit (SSU) rRNA gene was amplified using the primer pair 926f/1392r as described in Kunin et al. (2010). The reverse primer included a 5-bp barcode for multiplexing of samples during sequencing. Sequencing of PCR amplicons was performed at the Joint Genome Institute (JGI) using Roche 454 GS FLX Titanium technology following manufacturer’s instructions with the exception that the final dilution was 1*e*^-8^ (Allgaier et al., 2010). Of the 64 total samples, one of the Kansas native prairie samples did not sequence properly, yielding 63 samples for bioinformatics and statistical analysis.

### Lipid Analysis

Each core sample was homogenized and a 6-g portion was frozen at -20°C prior to lipid extraction. Membrane lipids were extracted from 3-g lyophilized and milled material in a two-phase aqueous-organic extraction (Bligh and Dyer, 1959). FAME analysis was conducted as described by Microbial ID (Kunitsky et al., 2006). Lipid methyl esters were determined using a Hewlett-Packard 6890 Gas Chromatograph configured and maintained for lipid analysis according to the recommendations of MIDI (Kunitsky et al., 2006). Gas chromatogram parameters were specified and peaks were identified by the MIDI EUYKARY method (MIDI, Newark, DE, United States). Fatty acid concentration was quantified by comparisons of peak areas of the samples compared to two internal standards, 9:0 (nonanoic methyl ester) and 19:0 (nonadecanoic methyl ester) (Sigma, St. Louis, MO, United States), of known concentration. In all subsequent analyses, we excluded fatty acids that were at an average abundance of <0.5 mol% or present in <3 samples.

Total abundance of lipids was used as an index of total microbial biomass. The abundance of indicator lipids for Gram-negative and Gram-positive bacteria, Actinobacteria, and saprophytic and arbuscular mycorrhizal fungi were further analyzed to indicate community response to treatment variables (Vestal and White, 1989; Balser and Firestone, 2005). Lipid data (mol%) were arcsine-transformed for normality and multivariate principal component analysis was carried out using JMP software, version 5.0.

### Bioinformatic and Statistical Analyses of 16S rRNA Gene Sequences

16S rRNA gene sequencing resulted in 646,884 16S rRNA gene reads, which were processed in QIIME 1.8.0 (Caporaso et al., 2010). Sequences were denoised, quality filtered, and chimera checked. Clustering was done at a 97% similarity threshold. Operational taxonomic units (OTUs) were assigned to sequences based on 97% identity using the open-reference USEARCH algorithm (Edgar, 2010). Finally, clusters were assigned OTU identifiers, resulting in 8,291 OTUs and a median of 9,776 sequences per sample. The recommended sampling depth of 3,266 sequences/sample for downstream analysis eliminated only one sample (Wisconsin restored prairie, 1 m south from core). OTUs were assigned taxonomy against the May 2013 release of GreenGenes (Lawrence Berkeley National Laboratory, Berkeley, CA, United States) using the RDP classifier method in QIIME.

### Alpha Diversity

Alpha and beta diversity metrics were calculated in QIIME. Multiple rarefactions were performed on the OTU table with a minimum and maximum number of sequences of 200 and 3,200, respectively, and a step-size of 500 and 100 iterations, producing 600 rarified tables. Alpha diversity was calculated on the rarified tables using the phylogenetic whole tree method. Rarefaction plots were generated from collated alpha diversity files and each metadata category was plotted. Finally, a student’s *t*-test was used as implemented in the QIIME script compare_alpha_diversity.py to perform pairwise comparisons between the alpha diversity values of samples of a given metadata category.

To calculate alpha diversity by state when considering only corn and native prairie samples, an OTU table was filtered to exclude the restored prairie and switchgrass samples. Multiple rarefactions were performed on the resulting OTU table with a minimum and maximum number of sequences of 500 and 4,500, respectively, and a step-size of 500 and 50 iterations, producing 400 rarified tables. Alpha diversity was subsequently calculated on the rarified tables using the phylogenetic whole tree method as described above. A student’s *t*-test was used as described above to calculate pairwise comparisons of alpha diversity by state.

### Beta Diversity

Beta diversity was estimated by calculating unweighted UniFrac distances and visualized using principal coordinate analysis. The PERMANOVA test embedded within the QIIME software suite was used to determine the degree to which categorical metadata parameters explained patterns in the UniFrac distance matrix (permutations = 999).

Because our data are not normally distributed, we used the non-parametric Kruskal–Wallis and Mann–Whitney tests implemented in QIIME on the single rarified table to determine which taxa were significantly different in abundance than expected if the OTUs were randomly distributed in the samples. The Mann–Whitney test was used to compare which families differentiate corn and native prairie samples. Taxa with Bonferroni-corrected *P*-value less than or equal to 0.05 were chosen for visualization. The Kruskal–Wallis test was used to determine which families were significantly different among all four management practices (corn, switchgrass, native prairie, and restored prairie). Similarly, taxa with Bonferroni-corrected *P*-values less than or equal to 0.05 were selected for visualization.

### Correlation of Phylogenetic Profiles and Chemical Metadata

Spearman rank coefficients comparing the relationships between taxa and soil chemical metadata were calculated in R statistical software [R version 3.0.2, Comprehensive R Archive Network (CRAN)]. Briefly, a matrix containing chemical metadata was merged with a matrix containing relative taxonomic abundance, such that the Spearman rank coefficient was calculated between sample-matched chemical metadata and OTU abundance data summarized at the order level. To investigate relationships between bacterial taxa and lipid profiles and to compare the two methods, Spearman rank coefficients were similarly calculated between sample-matched lipid profiles and the relative abundance of OTUs summarized at the order level. Resulting correlation matrices were filtered such that only columns and rows containing at least one correlation ≥ an absolute value of 0.60 were retained. Heatmaps were produced in R statistical software using heatmap.2 (R version 3.0.2, CRAN).

### Metagenomic Sequencing, Assembly, and Annotation

We performed shotgun metagenome sequencing using the reference core (0 m) DNA from the native prairie and continuously cultivated sites in each state. Libraries with ∼270 bp inserts were generated with the Illumina TruSeq protocol. Sequencing for each sample was conducted over a period of more than a year and spanned several platform improvements. As a result, for each sample sequence data was a mixture of Illumina GA2 (2 × 76 bp), GAIIx (2 × 100 bp, 2 × 114 bp, and 2 × 150 bp), or HiSeq 2000 (2 × 100 bp). Sequence data were deposited into the NCBI Short Read Archive (**Supplementary Table S2**).

Due to the large number of reads in these datasets, a Convey (Richardson, TX, United States) HC-1 hybrid core computer was used for preprocessing and roadmap construction. Graph phases of assembly used the Convey implementation (cnygc version 2.0.3208) of Velvet and were run either on the HC-1, a Sun Fire X4600 M2 with 1TB of RAM, a Dell R910 with 1TB of RAM or an IBM 3850 with 1 TB of RAM. Read pre-processing included trimming reads of Illumina quality ‘B’ using the cnygc–trimB operation. Velvet version 1.2.03 was used for contig construction (Zerbino and Birney, 2008). Details for each assembly are included in the **Supplementary Methods**. Sequencing and assembly statistics are summarized in **Supplementary Table S3**. Assembled contigs were submitted to Integrated Microbial Genomes (IMG) metagenome annotation pipeline for gene calling and annotation (Markowitz et al., 2014; Huntemann et al., 2015). Predicted protein sequences from IMG were compared to the FOAM database (Prestat et al., 2014) using hmmsearch at default settings (Eddy, 2011). For every sample, the relative abundance of each gene was calculated by dividing the number of hits to that gene by the total number of hits to the FOAM database.

### Core Functional Analysis

We defined core genes as those having similar abundances across communities (Shade and Handelsman, 2012). Rare gene families (those observed less than 100 times across all samples) were removed from analysis. Rank abundance curves were generated for each sample and the variance in rank abundance across samples was calculated for each gene. Genes varying the least in rank abundance were considered to represent core genes. To identify functional categories enriched in core genes, we counted the number of core genes (defined as the top 10% of genes varying the least in rank abundance) in each category of the second functional sublevel of the FOAM hierarchy. Permutation tests were performed by randomly assigning different outcome variances to each gene from the observed set of variances 10,000 times to obtain 95% confidence intervals for the number of core genes expected in each category. *P*-values were corrected for multiple testing using the false discovery rate (Benjamini and Hochberg, 1995).

### Phylogenetic and Taxonomic Analysis of Nitrous Oxide Reductase (nosZ)

Phylogenetic diversity of denitrifiers was investigated using the 795 *nosZ* sequences identified in contigs by IMG/M. To perform multiple sequence alignments, we built a custom HMM profile using *nosZ* amino acid sequences downloaded from the functional gene pipeline & repository (FunGene) database (Fish et al., 2013) that exceeded 1100 amino acids in length and had a minimum score of 630. The HMM profile was built from the FunGene seed alignment using hmmbuild from the HMMER3 package at default settings (Eddy, 2011). *NosZ* sequences from our dataset were aligned using hmmalign at default settings. Confidence scores were assigned to each alignment position using Zorro (Wu and Scott, 2012). Residues scoring less than 0.01 were removed from the alignment for tree building purposes. Because genes predicted from metagenome assemblies are often only partial sequences, many do not overlap. Therefore, we selected only sequences greater than 20 amino acids in length that overlapped the region with the highest alignment certainty as determined by Zorro scores. *NosZ* sequences curated and classified by Sanford R.A. et al. (2012) were added to the alignment using the muscle profile alignment algorithm (Edgar, 2010). A phylogenetic tree was inferred using FastTree (Price et al., 2009) at default settings. The tree was rooted using *Haloarcula marismortui* and *Halorubrum lacusprofundi* (Sanford R.A. et al., 2012). We assigned taxonomy to individual *nosZ* sequences by performing a BLASTP (Altschul et al., 1990) search against the National Center for Biotechnology Information non-redundant (NCBI-NR) database using an *E*-value cutoff of 1 × 10^-5^. The resulting file was imported into MEGAN, which performed taxonomic classification (Huson et al., 2016).

### Carbohydrate Active Enzymes

Glycoside hydrolase (GH) genes were identified in the raw metagenomic sequence data by comparison to the Carbohydrate Active Enzyme (CAZy) database (Lombard et al., 2014) using the UBLAST algorithm within the USEARCH program^1^ with an acceleration value 0.2 and an *E*-value cutoff of 1 × 10^-5^. GH family assignments were made based on the top hit. We verified the assignments by comparing putative GH sequences to the NCBI-NR database using UBLAST with the same parameters described above.

## Results

### 16S rRNA Gene Sequencing and Diversity Analysis

We sampled native tallgrass prairie (NP) sites from three states (Kansas, Iowa, and Wisconsin) representative of the Midwestern United States (U.S.) prairie ecosystem. At each location, a nearby site was selected that experienced long-term cultivation and was planted to corn (CC) when sampled. At the Wisconsin site, adjacent switchgrass monoculture and restored prairie plots were available and were also sampled. Sequencing of the 16S rRNA gene yielded 645,542 high quality sequences and identified 8291 OTUs.

Computation of alpha diversity metrics revealed significant differences in richness and phylogenetic diversity between cultivated and native prairie sites (**Supplementary Figure S2** and **Supplementary Table S4**). Alpha diversity was significantly higher overall in cultivated soils compared to native prairie soils (*P* = 0.006) and in switchgrass compared to native prairie soils (*P* < 0.04), although within-state alpha diversity metrics were not significantly different between management practices (e.g., Iowa CC versus Iowa NP). Alpha diversity was not different between the 250 mg sample taken from the soil core (10 g) and the large scale (500 g) samples, indicating ability to resolve complexity was already saturated with small sample sizes. Alpha diversity differed significantly among states, with Kansas having the highest alpha diversity and Iowa the lowest. All state pairwise comparisons were significant (*P* < 0.05).

**FIGURE 1:**
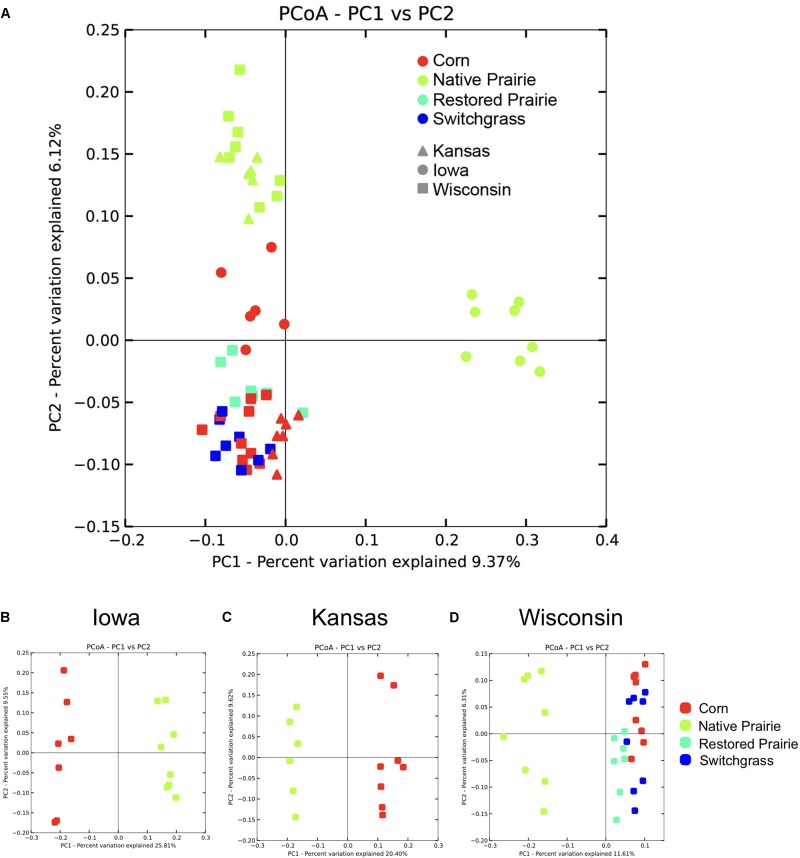
Visualization of beta-diversity reveals clustering by management type. Unweighted Unifrac distances were plotted using Principal Coordinate Analysis (PCoA) in QIIME. Each point represents a discrete sample. **(A)** PCoA plots of all sites and samples. **(B–D)** PCoA plots by state.

Beta diversity analysis showed clustering of soil samples by management practice across the data set (**Figure 1A**) and within states (**Figures 1B–D**). Bacterial communities from cultivated soils clustered together but were separate from native prairie communities (**Figure 1A**). When comparing samples by state, native prairie, and corn samples formed discrete clusters (**Figures 1B–D**). At the Wisconsin site, switchgrass, and restored prairie locations were also sampled and the microbial communities in all of the plots that had been cultivated exhibited considerable overlap (**Figures 1A,D**). State, site, and land management were each found to be significant factors that explained UniFrac distances (*P* < 0.05). Site was the strongest factor in determining community differences (Pseudo-F 3.814, *P* = 0.001), closely followed by management practice (Pseudo-F 3.811, *P* = 0.001). Distance within sites (0, 1 cm, 1 m, and 10 m) was not significant.

### Potential Bioindicators of Land-Management Practice and Soil Chemistry

**FIGURE 2:**
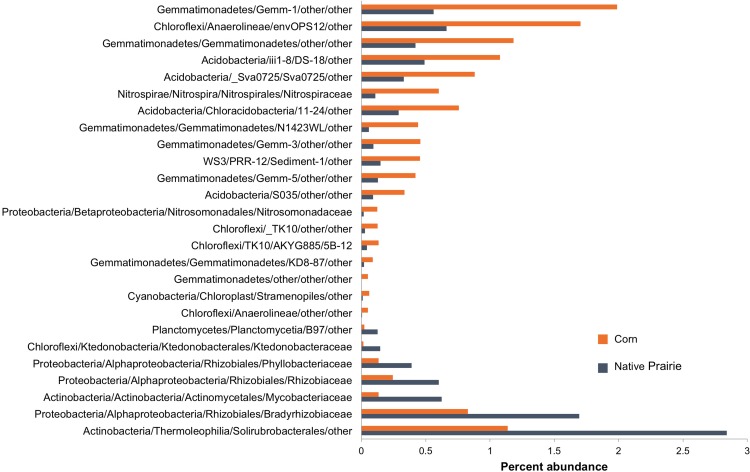
Abundance of bacterial families that differentiate corn and native prairie samples. Key OTUs at the family level that are significantly different between corn and native prairie samples, regardless of state (Kansas, Wisconsin, and Iowa). The non-parametric Mann–Whitney *t*-test (number of permutations = 999) was used to compare relative abundance of families in corn and native prairie samples from a single-rarified OTU table at 3,266 sequences/sample. Families with Bonferroni corrected *P*-values <0.05 were chosen for visualization.

Twenty-six microbial families differed significantly in abundance between corn and native prairie samples (*P* < 0.05, **Figure 2** and **Supplementary Figure S3**). Taxa that were more abundant in the cultivated corn soils included the families *Nitrosomonadaceae*, *Nitrospiraceae*, three unknown families of class *Gemmatimonadetes*, and two unknown families of class *Anaerolineae*. Only seven families were significantly more abundant in native prairie samples, including *Rhizobiaceae*, *Phyllobacteriaceae*, *Bradyrhizobiaceae*, *Mycobacteriaceae*, and *Ktedonobacteraceae*. Many of these families have members that carry out key nitrogen cycle processes. The shift from nitrogen fixers in prairie soils to those capable of nitrification in cultivated soils is presumably a response to nitrogen fertilizer application.

Spearman correlations between bacterial orders and soil chemical metadata revealed significant trends, specifically in relation to nitrogen (**Supplementary Figure S4**). *Rhizobiales* were strongly correlated to ammonium (NH_4_^+^) (ρ = 0.61, *P* < 0.001), while the putative orders *Gemm.5*, *Gemmatimonadetes N1423WL*, and *Acidobacteria Sva0725* were negatively correlated to ammonium (ρ = -0.67, -0.61, and -0.61, respectively, *P* < 0.001).

### Lipid Profiles

**FIGURE 3:**
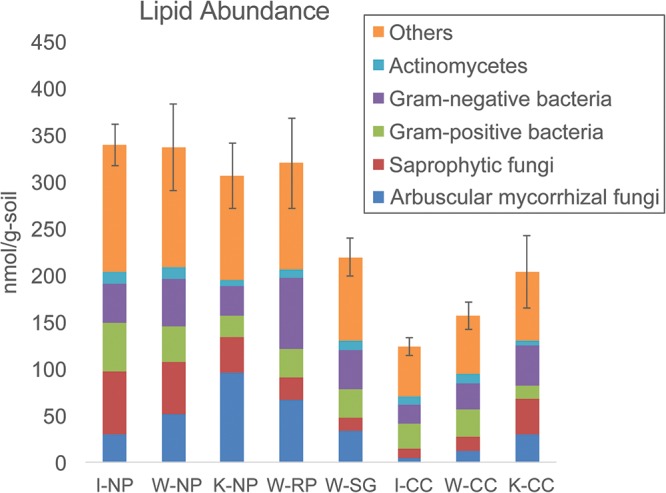
Total lipid abundance (microbial biomass nmol/g) and distribution patterns (%) of the main microbial groups. “Others” indicates microbial groups where taxonomic origin cannot be determined. Samples are (from left to right): Wisconsin native prairie, Kansas native prairie, Wisconsin restored prairie, Wisconsin switchgrass, Iowa cultivated corn, Wisconsin cultivated corn, and Kansas cultivated corn. Bar graphs show total lipid abundance and the relative abundance of each group. Error bars are the standard error of the total lipid abundance.

Lipid analysis indicated higher microbial biomass in the native prairie soils compared to the cultivated corn soils, mainly due to lower fungal abundances associated with cultivated corn soils (**Figure 3** and **Supplementary Tables S5**, **S6**). Arbuscular mycorrhizal fungi, saprotrophic fungi, protozoa, and *Actinobacteria*-associated lipids were also higher in abundance in native prairie soils than in cultivated corn soils. Both Gram-negative and Gram-positive associated lipids were more abundant in native prairie soils, although the Gram-negative to Gram-positive ratios were not significantly different (**Supplementary Figure S5**). Notably, 37 of the 50 measured lipids were significantly more abundant in native prairie soils, while only four lipids were slightly, but not significantly, greater in the cultivated corn: 16:1 Cis Alcohol w7, 16:1 ISO G, 16:1 w7c, 18:0 2OH (**Supplementary Table S6**).

There was a significantly higher ratio of fungal to bacterial lipids in Kansas native prairie compared to the other sampling locations (**Supplementary Figure S5**). The ratios of lipids corresponding to arbuscular mycorrhizal fungi compared to saprotrophic fungi were consistently lower in cultivated corn, and significantly higher in Kansas native prairie, Wisconsin switchgrass, and Wisconsin restored prairie (**Supplementary Figure S5**). This suggests that the grasses in these specific fields may be more effectively colonized with symbiotic fungi.

Spearman correlations of lipid profiles to relative abundances of bacterial orders revealed significant relationships between particular taxa and lipids (**Supplementary Figure S6**). *Rhizobiales, Ktedonobacterales*, and *Planctomycetia order B97* were positively correlated with most measured lipids. Notably, families belonging to these bacterial orders were differentially abundant in corn and native prairie soils, with increased abundances in native prairie soils (**Figure 2**). Conversely, orders belonging to the *Gemmatimonadetes* class, *Nitrosomonadales*, and *Anaerolineae* order envOPS12 were negatively correlated with most measured lipids (**Supplementary Figure S6**). Likewise, families of these orders are differentially abundant between corn and native prairie soils, with elevated abundance in the former (**Figure 2**). Together, these trends are congruent with the general observation of higher microbial biomass in native prairie samples compared to cultivated samples.

### Core Functional Gene Analysis

We performed metagenome sequencing on reference cores from the native prairie and cultivated corn soils in each state. Metagenome sequencing resulted in 1.3 terabases (Tb) of sequence data from the six samples (ranging from 159 to 327 Gb per sample). *De novo* assembly was performed on each of the sequence datasets, yielding 50.8 million contigs >200 bp in length totaling 16.8 Gb of assembled data (**Supplementary Table S3**).

To explore the functional gene repertoire in our samples, we refrained from between site comparisons because of lack of within site replication. Instead, we identified core functions shared across all samples (**Supplementary Table S7**). We defined core genes as those varying least in abundance across all sites. These genes may form a backbone supporting ecosystem processes critical in both native and cultivated soil communities. Conceptually, this is similar to taxonomically based core microbiome analyses discussed in Shade and Handelsman (2012). Among the top core genes, we found genes involved in transport, cell regulation and signaling, and nitrogen metabolism. One of the top core genes was adenylate cyclase, which is an essential part of microbial cyclic AMP (cAMP) signaling. Genes related to cAMP signaling have been observed at high frequency in other soil metagenome surveys (Delmont et al., 2012). Two serine/threonine protein kinases, which are widely distributed across bacterial and archaeal phyla and play an important roles in physiology, regulation of cells division and translation, and environmental sensing (Pereira et al., 2011; Shi et al., 2014), were also among the top core genes. Nitrite reductase (nirK), a nitrogen regulatory protein C (ntrC) family gene, and two NitT/Tau family ABC transport genes were among the core genes related to the nitrogen cycle.

To formally identify functional groups enriched in core genes, we combined genes into FOAM ontological groups and found that at functional level 1 (the most general functional level), the prokaryotic type ABC transporters (*P* = 0.013) and regulation of response to osmotic stress (*P* = 0.047) categories had more core genes than expected under the null hypothesis that core genes are randomly distributed across FOAM functional categories. Within the prokaryotic type ABC transporters category, the genes detected encoded transporters of a broad range of compounds, including sugars, amino acids, peptides, cell wall components, and metals (**Supplementary Figure S7**). In the “regulation of response to osmotic stress” FOAM category, genes detected included those encoding sensor kinase proteins of the two-component signal transduction system, two of which belonged to the ompR family (KO: K07636; K02484) that allow bacteria to sense changes in osmolarity (Feng et al., 2003).

### Nitrogen Metabolism

**FIGURE 4:**
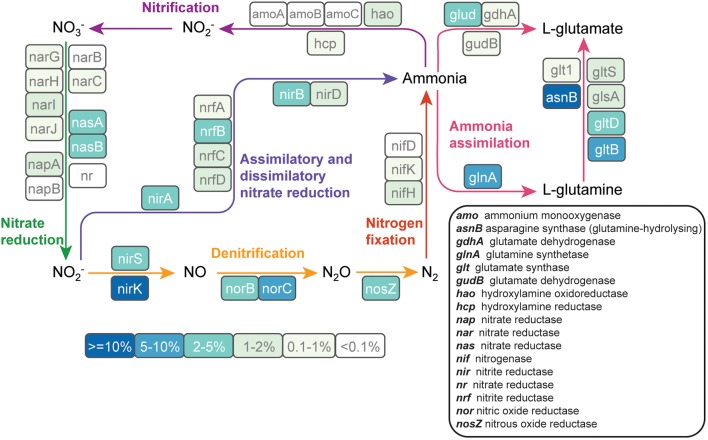
The abundance of nitrogen cycle genes in cultivated and never-cultivated tall grass prairie soils in the Midwestern United States. Each gene in the nitrogen cycle is enclosed in a colored box. The color of the box indicates the abundance of each gene relative to all nitrogen cycle genes in the assembled metagenome data. Genes were identified on contigs by comparing predicted protein sequences to the FOAM database. Percentages are averaged across all samples.

Because nitrogen fertilization is a major perturbation to cultivated soils and nitrogen is a key driver of soil microbial community composition (Fierer et al., 2012, 2013), we analyzed nitrogen metabolism pathways that were reconstructed from metagenomic sequence data. Forty-nine genes involved in all major components of the nitrogen cycle were detected across the metagenomes (**Figure 4**). Genes involved in denitrification were more abundant than nitrification genes. Those involved in the conversion of nitrite to nitrogen gas (*nirK*: 18%*, nirS:* 3.1%*, norC*: 6.5%*, norB*: 2.0%, and *nosZ*: 3.4%) made up 33% of all detected nitrogen cycle genes. Genes involved in ammonia assimilation accounted for 39% of all the nitrogen cycle genes. Ammonia monooxygenase, a key gene in the nitrification pathway, was only detected at low levels (<0.3% of nitrogen-cycle genes). *NifH*, the key marker gene for nitrogen fixation, accounted for 1.7% of the nitrogen-cycle genes.

**FIGURE 5:**
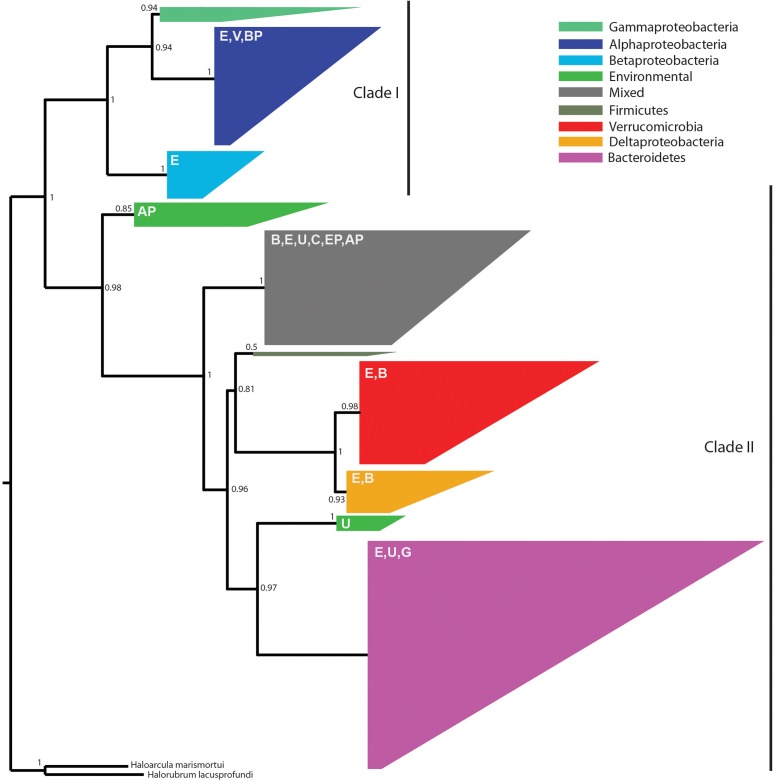
An approximate maximum-likelihood phylogenetic tree of 538 *nosZ* amino acid sequences predicted from metagenome assemblies and 69 sequences previously characterized as typical (Clade I) or atypical (Clade II) (Sanford G.R. et al., 2012). Reliability of each split in the tree was calculated using the Shimodaira–Hasegawa test. The tree was rooted using *Haloarcula marismortui* and *Halorubrum lacusprofundi* (Sanford R.A. et al., 2012). Clades are colored according to the majority taxonomic group hosting the majority of sequences. Letters in the upper left of each clade indicate the presence of secondary taxa. E, environmental; V, Verrucomicrobia; BP, Betaproteobacteria; AP, Alphaproteobacteria; B, Bacteroidetes; U, unassigned; C, Chloroflexi; EP, Epsilonproteobacteria; G, Gemmatimonadetes. One group, referred to as mixed in the figure legend, contained no dominant taxonomic group.

Because the denitrification pathway was highly represented in the metagenomes, we focused on the phylogenetic diversity of denitrifiers. Specifically, we focused on *nosZ* sequences because NosZ is the only enzyme known to catalyze the last step of denitrification: conversion of nitrous oxide (N_2_O) to nitrogen gas (N_2_) whereas other steps in the denitrification pathway can be catalyzed by multiple enzymes (Jones et al., 2008). Phylogenetic analysis revealed two distinct clades with strong bootstrap support corresponding to typical (Clade I) and atypical *nosZ* (Clade II) genes (**Figure 5**). We were able to assign taxonomy using the lowest common ancestor algorithm (Huson et al., 2016) to 80% of the Clade I sequences at the phylum level, the majority of which were from *Proteobacteria.* Genes with higher resolution taxonomic assignments were affiliated with *Alphaproteobacteria*, *Betaproteobacteria* (primarily *Burkholderiales*), or *Gammaproteobacteria* (primarily *Pseudomonadales*). The 20% of the sequences not assigned to a specific taxonomic group were classified as environmental sequences.

Among the two-thirds of *nosZ* sequences from this study that clustered with Clade II *nosZ* genes, we were able to classify approximately 75% at the phylum level; the remaining 25% were either environmental or unassigned. We found greater taxonomic diversity among Clade II *nosZ* genes and they were primarily affiliated with *Bacteroidetes* (41%), *Verrucomicrobia* (18%), *Deltaproteobacteria* (7%), and at lower levels to *Chloroflexi, Epsilonproteobacteria, Gemmatimonadetes, Alphaproteobacteria, Betaproteobacteria*, and *Firmicutes* (**Figure 5**).

### Carbohydrate Metabolism

We explored the repertoire of carbohydrate-degrading enzymes in the soil communities by comparing raw reads to GH sequences from the CAZy database (Lombard et al., 2014). The most dominant GH gene families were GH13, representing 36% of all GH sequences, and GH15 at 8% (**Supplementary Table S8A**). Glucoamylases, which are involved in starch hydrolysis, constituted the bulk of the GH15 enzymes (Cantarel et al., 2009; Marín-Navarro and Polaina, 2011). GH13 also contained a large number of starch-degrading enzymes. Taken together, these data suggest high amylolytic (i.e., starch-degrading) potential in the sampled soil region.

Glycoside hydrolase families targeting plant structural polysaccharides were categorized by function (Pope et al., 2010; Hess et al., 2011) and evaluated separately from other GH families because of their potential to decompose recalcitrant biomass. Endoglucanases (cellulases) were primarily represented by families GH5 and GH9; these two GH families accounted for approximately 9% of all reads within the plant structural polysaccharides category. Other cellulase families were detected at low levels (<1.5%; **Supplementary Table S8B**). β-glucosidases, primarily GH1, accounted for ∼10% of the plant polysaccharide degrading genes; sequences predicted to be xylanases (GH10 and GH11) made up another 5%. The primary hemicelluloses found in grass cell walls contain L-arabinose side chains (Scheller and Ulvskov, 2010), which may explain the high abundance of α-L-arabinofuranosidases (GH51, GH54, and GH62: 12.9–16.8%) in all of the samples examined.

## Discussion

The former tallgrass prairie region of the Midwestern United States is an area of economic and ecological importance for food security, biofuel production, nutrient retention, and is a major terrestrial carbon store, that could be jeopardized with climate change (Boody and DeVore, 2006; Jordan and Warner, 2010; Jokela et al., 2011; Paustian et al., 2016). Predicting the environmental consequences of changes to prairie-derived soils resulting from cultivation practices will likely be improved by understanding the microbial communities involved in carbon and nutrient cycling before and after cultivation.

Here we performed lipid profiling of microbial biomass as an indicator of soil metabolic health and quality (Vestal and White, 1989; Yao et al., 2000). We observed approximately double the microbial (lipid) biomass in native prairie samples compared to their paired cultivated samples (**Figure 3** and **Supplementary Table S6**), suggesting that the prairie is more supportive of microbial biomass likely resulting from higher levels of soil carbon. This observation agrees with the results of Spearman rank correlations of taxa to lipid abundances, in that taxa positively correlated with cultivated corn samples were negatively correlated to total abundance of most measured lipids. Conversely, taxa positively correlated with native prairie samples, such as families of the *Rhizobiales* order, showed strong positive correlations to most measured lipids (**Supplementary Figure S6**). The abundance of arbuscular mycorrhizal fungi in native prairie is not surprising as several of the tallgrass prairie grasses are known to be mycorrhizal dependent (Wilson and Hartnett, 1998; Hoeksema et al., 2010; van der Heijden et al., 2015; Koziol and Bever, 2016).

Although the biomass was higher in prairie, alpha diversity was significantly higher in cultivated sites; cultivated corn showed the highest diversity, followed by switchgrass, restored prairie, and native prairie. Evenness did not differ significantly between prairie and cultivated corn. This finding is consistent with the work of Barber et al. (2017) who found that alpha diversity was lowest in native prairie and long-term restoration sites compared to agriculture fields and recently restored sites. Similarly, Acosta-Martinez et al. (2008) found elevated alpha diversity in samples from cultivated cornfields. This increase in alpha diversity, but with a much lower biomass, may be the result of agricultural practices associated with cultivation that provide more microbial niches, such as application of nitrogen-based fertilizer and/or the higher annual fluxes of organic carbon turnover stemming from plant productivity and litter input. Ample provision of otherwise scarce nutrients, such as nitrogen and phosphorus could drive fertilizer-associated increases in diversity (Acosta-Martinez et al., 2008; Mao et al., 2011). Notably, all of the corn plots in our study received a nitrogen-based fertilizer.

Long-term cultivation also resulted in significant changes in bacterial community structure. The soil microbiomes exhibited distinct clustering according to land management practice, with cultivated samples clustering together and separately from the native prairie samples, suggesting evidence of a cultivation-specific microbiome. The native prairie samples not only clustered distinctly from the cultivated samples but also clustered separately by state, suggesting that a combination of local soil history, plant species, and climate influence soil microbial community structures. The similarity of cultivated soil communities–whether from corn, switchgrass (3 years since cultivation), or even restored prairie (10 years since cultivation)–suggests that cultivation in general has a profound influence on the microbial community structure independent of crop species. In a similar vein, Fierer et al. (2012) found that a common practice in cultivation–high levels of N input–changed community structure similarly in both monoculture and grassland sites compared with low and intermediate levels of N addition (Fierer et al., 2012).

The observation that the restored prairie samples have bacterial communities that are intermediate between prairie and cultivated locations (**Figure 1D**), but more similar to those from the long-term cultivated sites is notable. This finding suggests that the changes associated with agricultural practices endure over long time periods, and that the return of the soil microbiome to the composition found in the native prairie state is a slow process. This is in agreement with previous findings of no difference in bacterial community composition between traditionally managed agricultural fields and a previously cultivated field that was left to recover for 9 years (Buckley and Schmidt, 2003) but in contrast with other data that suggest a faster time-frame for recovery (Herzberger et al., 2014). It is not clear what is driving the differences between these investigations, but it highlights the necessity of further studies to determine of how environment, vegetation, soil physicochemistry, and microbial processes interact in response to land-use changes.

Land-use management not only explained differences in microbial diversity and biomass, but also was correlated with taxonomic changes. In contrast to the native prairie samples, cultivated soil showed significantly higher abundances of the *Nitrospiraceae* and *Nitrosomonadaceae* families. The former is involved in ammonia oxidation to nitrite, the rate-limiting step in nitrification (Kowalchuk et al., 2000; Webster et al., 2005), while the latter oxidizes nitrite to nitrate (Lücker et al., 2010). The increased abundance of ammonia oxidizers in the cultivated soils may be a response to the application of ammonia-nitrogen fertilizer for production of corn. Nitrification activity is known to increase with nitrogen fertilizer application (Carey et al., 2016; Ouyang et al., 2016). In native prairie soils, several members of the order *Rhizobiales*–common rhizosphere-associated microbes–were more abundant than in cultivated soils. In contrast to recent studies (Fierer et al., 2013; Barber et al., 2017), we did not observe significant differences in *Verrucomicrobia* across sites or treatments.

While 16S rRNA gene sequencing revealed differences in taxa known to perform nitrification (cultivated corn) and nitrogen fixation (native prairie), analysis of the metagenomic data suggests that denitrification was uniformly important in both cultivated and native ecosystems, similar to observations in Nelson et al. (2016). Denitrification returns nitrogen to the atmosphere as inert N_2_ (complete denitrification) or the potent greenhouse gas N_2_O (incomplete denitrification). The most abundant nitrogen cycle gene (*nirK*, which reduces NO_2_^-^ to NO) encodes one of the first steps in this pathway and was found to be a core functional gene. Clade I and Clade II *nosZ* genes (encoding the final step in the denitrification pathway), were found in a large number of phyla from both native prairie and cultivated corn samples. Until recently, attenuation of soil N_2_O emissions was thought to be mediated primarily by members of the *Alpha*-, *Beta*-, and *Gamma*-*proteobacteria* that are capable of performing all steps in the denitrification pathway (Sanford R.A. et al., 2012). However, bioinformatics analyses have revealed phylogenetically distinct *nosZ* sequences (Clade II) in a diverse array of organisms lacking other genes in the denitrification pathway (Sanford R.A. et al., 2012). Our observation that approximately 2/3 of prairie soil *nosZ* genes were atypical corroborates a recent study surveying *nosZ* in different soil types (Orellana et al., 2014) and suggests a deep reservoir of phylogenetically diverse organisms capable of mitigating N_2_O emission through N_2_O reduction to N_2._

Soils represent one of the most complex microbial communities on Earth. As such, they present a unique challenge for assembling and analyzing metagenomic data. Prior analysis of the Iowa corn and Iowa prairie metagenomes demonstrated 48 and 31% of contigs (from corn and prairie, respectively) had coverage of less than 10 and that only ∼20% of the sequence data could be assembled (Howe et al., 2014). Full assemblies of soil metagenomes may require many terabases (Gans et al., 2005; Howe et al., 2014; Rodriguez-R et al., 2018). Because soils have substantial spatial heterogeneity, even down to the microstructure scale (Nesme et al., 2016), we designed the study to maximize sequence coverage of a small sample, i.e., to not dilute the community with extraneous DNA from different sites even though they might be local. This design for depth rather than breadth sacrificed the more traditional replicate design for the metagenome samples, limiting our ability to determine how functional genes differ between sites. Our 16S rRNA amplicon data, however, does provide replication for the sites.

Here, we focused on near-surface soils since they are more responsive to land management (Zhang, 2017). However, a significant proportion of biomass resides within deeper soils (Fierer et al., 2003) and deep-soil microbes contribute to long-term carbon sequestration (Rumpel and Kogel-Knabner, 2011). Depth is a major driver of community structure (Pereira et al., 2017; Zhang, 2017). Just as in surface soils, tillage (Sun et al., 2018), and soil physicochemical properties (Zhang et al., 2017) strongly affect microbial communities at depth. To gain a full understanding of how geographic distance, cropping systems, and long-term cultivation influence microbial community structure and function, future studies must consider both horizontal and vertical community distributions.

In summary, we found that cultivation has a significant impact on microbial community biomass, diversity, and composition in soils across the former tallgrass prairie region of the Midwestern United States. However, based on our metagenomic survey, we found that many core functions were conserved, even at small sample scales, across geographic regions and land management practices, suggesting that conditions common to prairie soil, independent of land-use, select for a set of critical features that persist despite perturbation. While DNA sequence information does not reflect current microbial activity since much of it may not be expressed at any given time, it does reflect microbial dynamics over a long time. The paired sites in this case had 50–100 years of cultivation versus none, which resulted in a major microbial biomass change (as documented by lipid data) and major microbial community change (as documented by 16S rRNA data). While the metagenomic portion of this study was designed to a sequence samples deeply because of soil community complexity, further studies are needed to sample more broadly to determine to what extent and which genes are selected under different land management and crop regimes.

## Author Contributions

RM, SK, and BP performed the metagenome analyses. AG performed the 16S analyses. RL supervised the bioinformatics. EJ assisted with the sampling and field data. AC performed the sequence data QC and metagenome assemblies. CL performed the lipid analyses and interpretations. RJ and CR the obtained the field samples. ST managed the sequencing and sequence analysis. JT and JJ conceived of study, managed sample preparation and data generation, and coordinated analyses. All authors assisted with writing of the manuscript.

## Conflict of Interest Statement

The authors declare that the research was conducted in the absence of any commercial or financial relationships that could be construed as a potential conflict of interest.

## Footnotes

1 http://drive5.com/usearch
