# Supplementary material for: Microbial Community Structure and Functional Potential in Cultivated and Native Tallgrass Prairie Soils of the Midwestern United States
Source: Front Microbiol. 2018 Aug 15;9:1775. doi: 10.3389/fmicb.2018.01775 (PMC6104126; doi:10.3389/fmicb.2018.01775)
Supplement: Supplementary file 1 [file Data_Sheet_1.docx]

**Supplemental Figures**

**
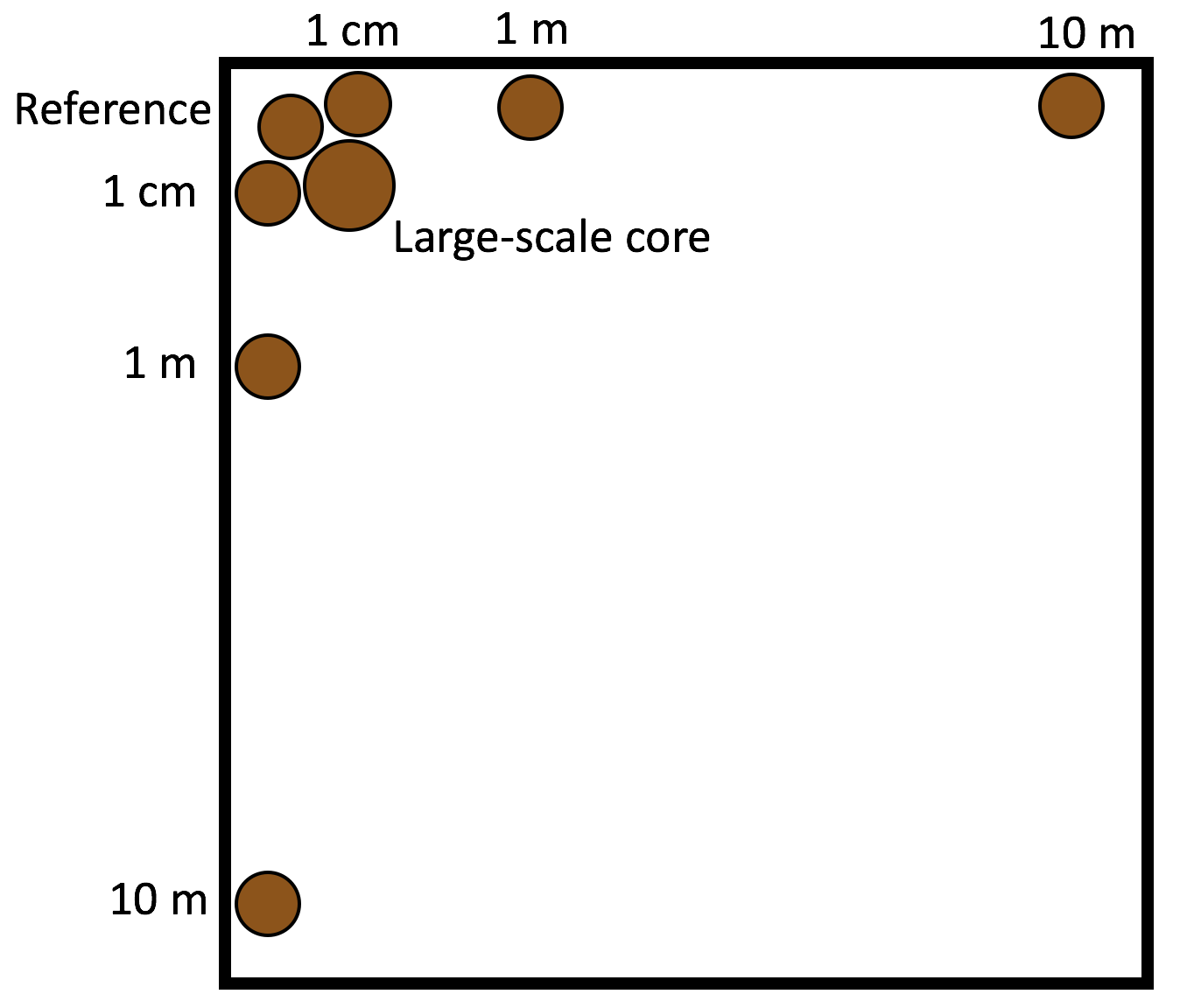
**

**Supplemental Figure 1**: Sample collection strategy. A reference (or apex core) was sampled and defined as 0 m. Six additional samples were taken at 90 degree angles from the reference at 1 cm, 1 m, and 10 m. A larger eighth samples was collected adjacent to the reference core.


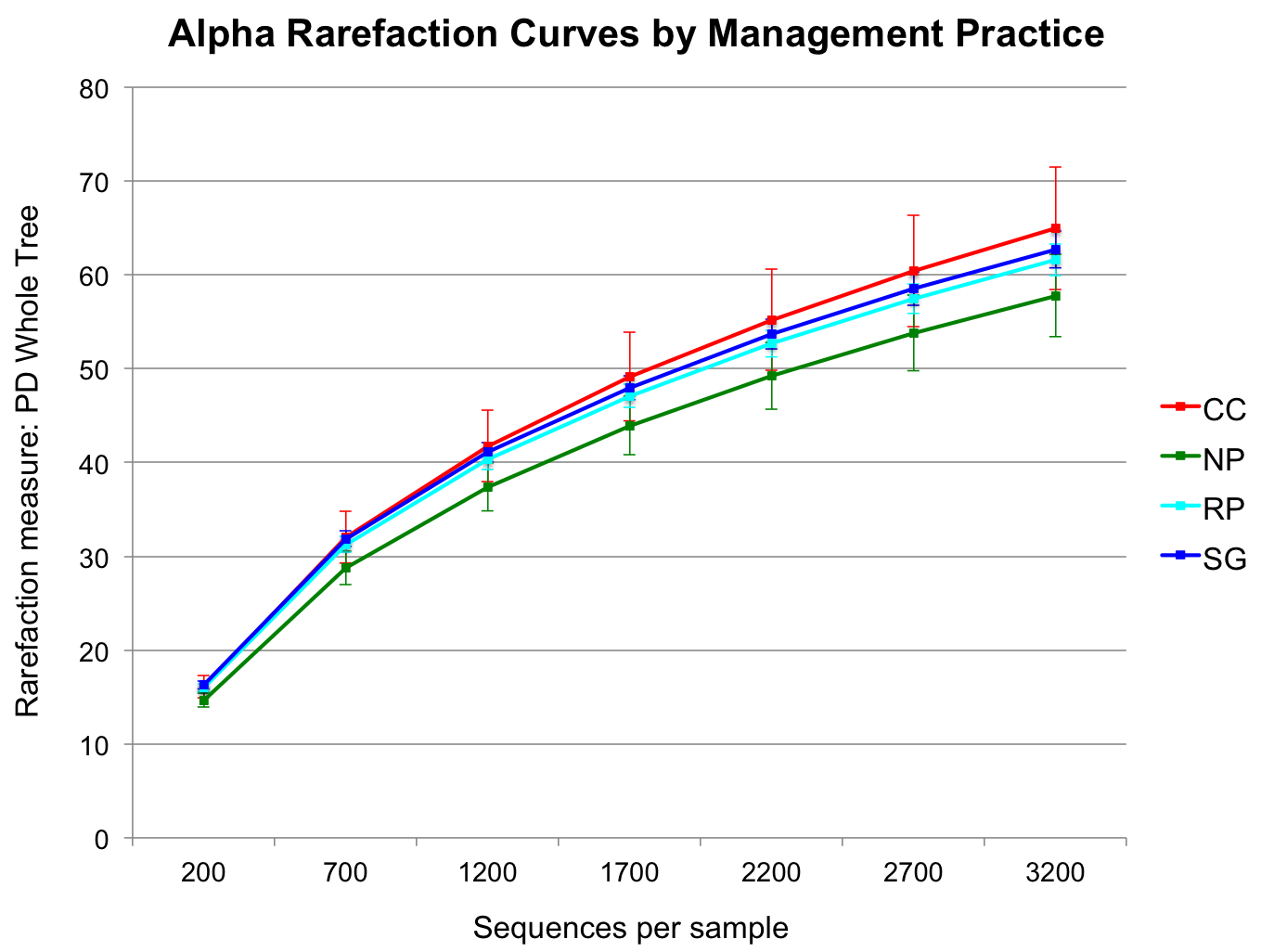


**Supplemental Figure 2**: Differences in alpha diversity are observed across management types. Multiple rarefactions were performed on the OTU table with a minimum and maximum number of sequences of 200 and 3200 respectively, and a step-size of 500 and 100 iterations, producing 600 rarified tables. Alpha diversity was calculated on the rarified tables using the phylogenetic whole tree method. Rarefaction plots were generated from collated alpha diversity files and plotted by management type. Alpha diversity was highest in corn samples, followed by switchgrass, restored prairie, and native prairie samples.


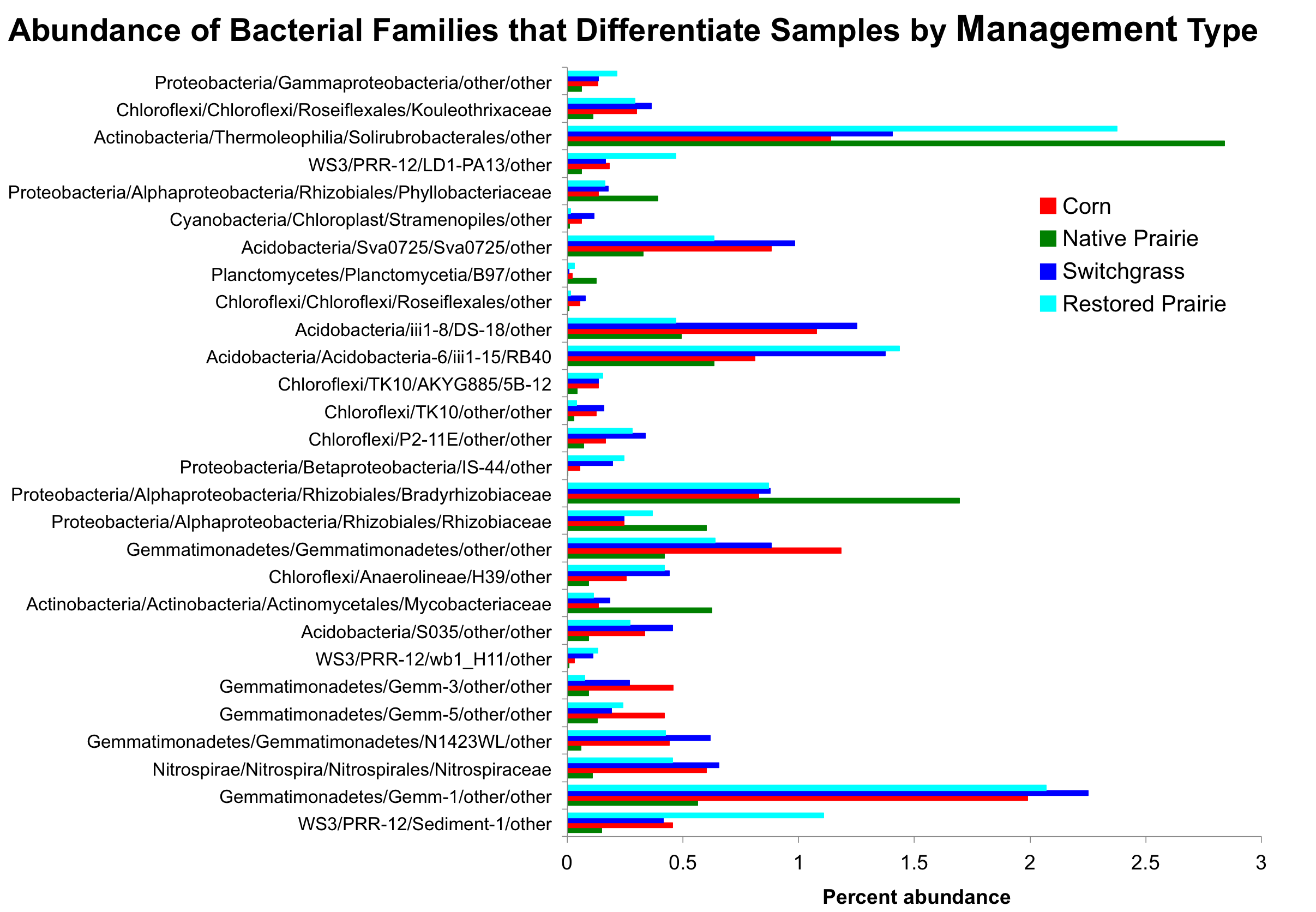


**Supplemental Figure 3:** Percent abundance of key bacterial families that significantly differentiate samples by management type, regardless of state (Kansas, Wisconsin, Iowa). The nonparametric Kruskal Wallis test (number of permutations = 999) was used to compare relative abundance of families by management type from a single-rarified OTU table at 3,266 sequences/sample. Families with Bonferonni-corrected p-values < 0.05 are represented. Relative abundance was converted to percent abundance for visualization.

**
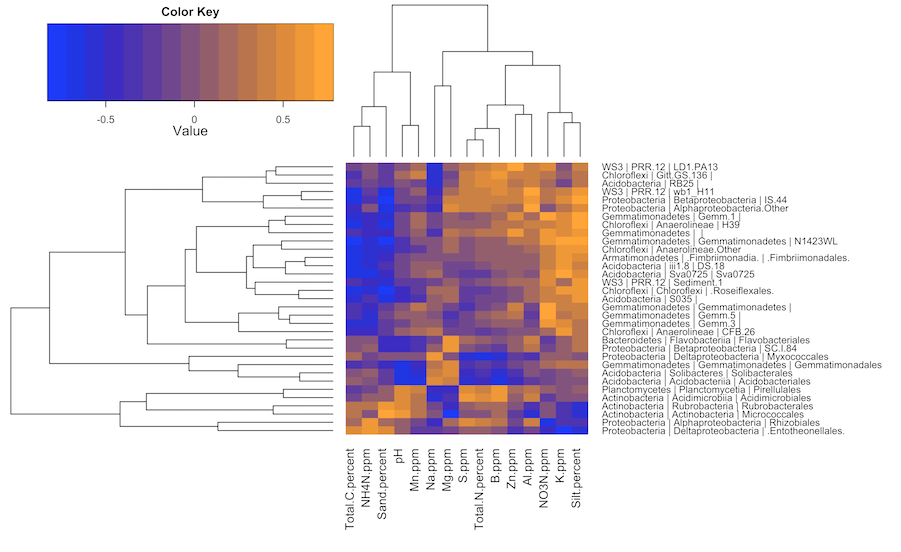
**

**Supplemental Figure 4:** Spearman rank coefficient correlations between sample-matched soil chemical parameters and bacterial orders reveal potential abiotic factors driving community differences. Spearman rank coefficients were calculated in R statistical software (R version 3.0.2, Comprehensive R Archive Network (CRAN)) between sample-matched chemical metadata and relative abundances of bacterial orders. The resulting correlation matrix was filtered such that only columns and rows with at least one correlation ≥ the absolute value of 0.60 were retained. Heatmaps were produced in R statistical software using heatmap.2 (R version 3.0.2, CRAN).


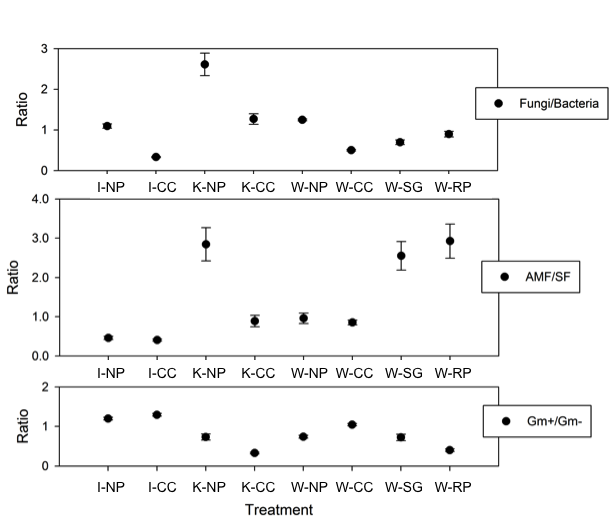


**Supplemental Figure 5:** Ratios of Fungi/Bacteria, Arbuscular mycorrhizal fungi (AMF)/Saprophytic fungi (SF), and Gram-Positive/Gram-Negative bacteria among sampled soil sites. The Kansas native prairie exhibited an exceptionally high fungi/bacterial ratio, consistent with its the elevated AMF/SF ratio. Ratios of Gram-Positive to Gram-Negative bacteria were not significantly different by management practice.

**
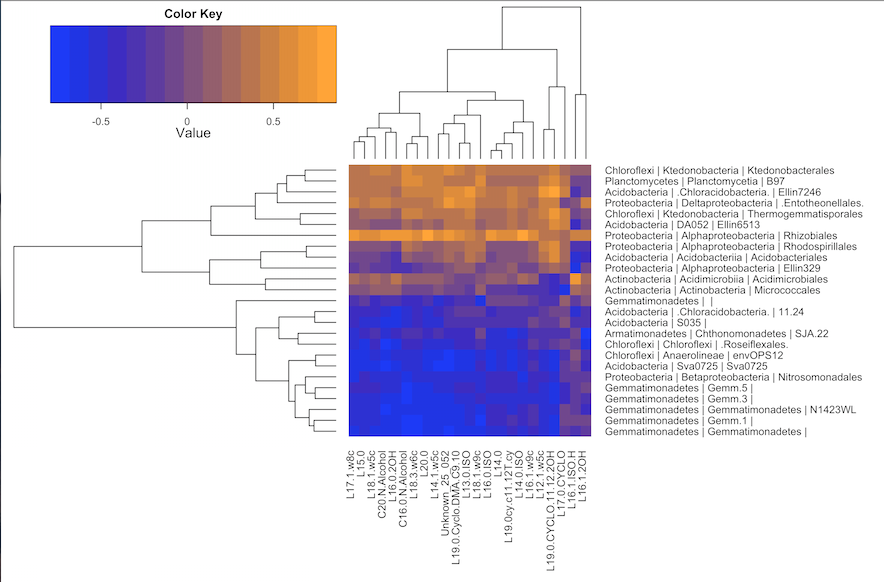
**

**Supplemental Figure 6:** Spearman rank coefficient correlations between sample-matched lipid profiles and bacterial orders. Spearman rank coefficients were calculated in R statistical software (R version 3.0.2, Comprehensive R Archive Network (CRAN)) between sample-matched measured lipid concentrations and relative abundances of bacterial orders. The resulting correlation matrix was filtered such that only columns and rows with at least one correlation ≥ the absolute value of 0.60 were retained. Heatmaps were produced in R statistical software using heatmap.2 (R version 3.0.2, CRAN).

**Supplemental Figure 7:** ABC transporters containing at least one subunit identified as a core gene. The subunits identified as core genes are colored. Subunits not identified as core genes are shown in grey.

**Supplemental Tables**

**Supplemental Table 1.** See excel spreadsheet

**Supplemental Table 2:** NCBI Short Read Archive accession numbers. C: cultivated, NP: native prairie, RP: restored prairie, SW: switchgrass.

| State | Kansas | | Iowa | | Wisconsin | | | |
| --- | --- | --- | --- | --- | --- | --- | --- | --- |
| Treatment | C | NP | C | NP | C | NP | RP | SW |
| Accession | SRP081636 | SRP081635 | SRP081657 | SRX099701 | SRX099538 | SRP081606 | SRP081568 | SRP081569 |
|  | SRP008742 | SRP008741 |  | SRX099700 | SRX099537 |  | SRP008668 |  |

**Supplemental Table 3.** Sequencing and assembly summary. 2x indicates paired-end sequencing. The number following 2x is read length. Data are total amount of sequence generated in gigabases (Gb). Count indicates the total number of reads or contigs generated. Sequence in contigs shows the total amount of sequence within the contigs in Gb.

|  |  | | Run Type | | | | | Assemblies | |
| --- | --- | --- | --- | --- | --- | --- | --- | --- | --- |
| **Sample** | **2x100** | **2x114** | | **2x151** | **2x76** | **Total** | **Read count** | **Contig count** | **Sequence in contigs** |
| IP | 267.99 | 24.25 | | -- | 34.76 | **327.00** | 6.44x10^13^ | 10,744,470 | 3.03 |
| IC | 141.61 | 35.35 | | 10.69 | 31.45 | **219.10** | 4.44x10^13^ | 2,716,529 | 0.92 |
| KP | 102.73 | 34.34 | | 15.51 | 6.61 | **159.19** | 2.70x10^13^ | 27,786,733 | 9.28 |
| KC | 173.95 | 53.24 | | -- | 13.50 | **240.69** | 4.90x10^13^ | 5,020,643 | 1.34 |
| WP | 165.38 | 28.34 | | -- | 2.96 | **196.68** | 4.00x10^13^ | 1,998,404 | 0.58 |
| WC | 176.69 | 13.04 | | -- | 2.40 | **192.13** | 3.87x10^13^ | 3,942,652 | 1.46 |

**Supplemental Table 4:** Alpha diversity metrics by sample location, state or by treatment type. Switchgrass and restored prairie samples were excluded from the Wisconsin samples to achieve a fair comparison including only corn and native prairie samples. Fisher’s alpha diversity was calculated by state, management practice, and sample location at the level of OTU, family, and phylum. Switchgrass and restored prairie samples were excluded from the state comparison to allow for comparison of only corn and native prairie samples by state.

| **Fisher’s Alpha** | | **OTU** | | | **Family** | | | **Phylum** | | |
| --- | --- | --- | --- | --- | --- | --- | --- | --- | --- | --- |
| **State** | **Seqs/Sample** | **Avg.** | **Error** | **P-value** | **Avg.** | **Error** | **P-value** | **Avg.** | **Error** | **P-value** |
| Iowa (IA) | 4500 | 525.07 | 80.96 | **vs. KS: 0.00** | 54.64 | 5.75 | **vs. KS: 0.00** | 3.85 | 0.43 | **vs. KS: 0.01** |
| Kansas (KS) | 4500 | 734.30 | 131.93 | **vs. WI: 0.05** | 64.82 | 4.46 | vs. WI: 0.17 | 4.46 | 0.48 | vs. WI: 1.00 |
| Wisconsin (WI) | 4500 | 615.56 | 102.13 | **vs. IA: 0.04** | 61.18 | 5.11 | **vs. IA: 0.01** | 4.38 | 0.36 | **vs. IA: 0.01** |
| **Management Practice** | **Seqs/Sample** | **Avg.** | **Error** | **P-value** | **Avg.** | **Error** | **P-value** | **Avg.** | **Error** | **P-value** |
| Cultivated corn (CC) | 3200 | 641.34 | 140.20 | **vs. NP: 0.02** | 64.28 | 5.22 | **vs. NP: 0.01** | 4.28 | 0.52 | vs. NP: 1.00 |
|  |  |  |  | vs. RP: 0.56 |  |  | vs. RP: 1.00 |  |  | vs. RP: 1.00 |
| Native Prairie (NP) | 3200 | 509.74 | 69.73 | vs. RP: 1.00 | 55.55 | 5.48 | vs. RP: 0.06 | 4.14 | 0.47 | vs. RP: 1.00 |
|  |  |  |  | vs: SG: 0.33 |  |  | vs: SG: 0.02 |  |  | vs: SG: 1.00 |
| Restored Prairie (NP) | 3200 | 547.24 | 46.81 | vs. SG: 1.00 | 61.68 | 1.35 | vs. SG: 0.64 | 4.18 | 0.23 | vs. SG: 1.00 |
| Switchgrass (SG) | 3200 | 566.33 | 60.33 | vs. CC: 1.00 | 63.28 | 2.04 | vs. CC: 1.00 | 4.18 | 0.40 | vs. CC: 1.00 |
| Sample location | **Seqs/Sample** | **Avg.** | **Error** | **P-value** | **Avg.** | **Error** | **P-value** | **Avg.** | **Error** | **P-value** |
| Iowa Corn | 3200 | 505.08 | 106.68 | vs. Iowa NP: 1.00 | 59.08 | 4.60 | vs. Iowa NP: 0.08 | 3.83 | 0.34 | vs. Iowa NP: 1.00 |
|  |  |  |  | vs: Kansas CC: 0.08 |  |  | vs. Kansas CC: 0.08 |  |  | vs. Kansas CC: 0.39 |
|  |  |  |  | vs. Kansas NP: 1.00 |  |  | vs. Kansas NP: 1.00 |  |  | vs. Kansas NP: 0.25 |
|  |  |  |  | vs. Wisc CC: 0.59 |  |  | vs. Wisc CC: 0.17 |  |  | vs. Wisc CC: 0.73 |
|  |  |  |  | vs. Wisc NP: 1.00 |  |  | vs. Wisc NP: 1.00 |  |  | vs. Wisc NP: 1.00 |
|  |  |  |  | vs. Wisc RP: 1.00 |  |  | vs. Wisc RP: 1.00 |  |  | vs. Wisc RP: 1.00 |
|  |  |  |  | vs. Wisc SG: 1.00 |  |  | vs. Wisc SG: 1.00 |  |  | vs. Wisc SG: 1.00 |
| Wisconsin Corn | 3200 | 633.39 | 75.17 | **vs. Iowa NP: 0.03** | 65.64 | 1.77 | vs. Iowa NP: 0.06 | 4.39 | 0.35 | vs. Iowa NP: 0.73 |
|  |  |  |  | vs. Kansas CC: 0.48 |  |  | vs. Kansas CC: 1.00 |  |  | vs. Kansas CC: 1.00 |
|  |  |  |  | vs. Kansas NP: 1.00 |  |  | vs. Kansas NP: 0.11 |  |  | vs. Kansas NP: 1.00 |
|  |  |  |  | vs. Wisc NP: 0.22 |  |  | **vs. Wisc NP: 0.03** |  |  | vs. Wisc NP: 1.00 |
|  |  |  |  | vs. Wisc SG: 1.00 |  |  | vs. Wisc SG: 0.90 |  |  | vs. Wisc SG: 1.00 |
|  |  |  |  | vs. Wisc RP: 1.00 |  |  | vs. Wisc RP: 0.08 |  |  | vs. Wisc RP: 1.00 |
| Kansas Corn | 3200 | 768.52 | 91.74 | vs. Iowa NP: 0.08 | 67.45 | 67.45 | **vs. Iowa NP: 0.03** | 4.56 | 0.55 | vs. Iowa NP: 0.67 |
|  |  |  |  | vs. Kansas NP: 0.14 |  |  | vs. Kansas NP: 0.67 |  |  | vs. Kansas NP: 1.00 |
|  |  |  |  | vs. Wisc SG: 0.08 |  |  | vs. Wisc SG: 1.00 |  |  | vs. Wisc SG: 1.00 |
|  |  |  |  | **vs. Wisc NP: 0.03** |  |  | vs. Wisc NP: 0.11 |  |  | vs. Wisc NP: 1.00 |
|  |  |  |  | **vs. Wisc RP: 0.03** |  |  | vs. Wisc RP: 0.59 |  |  | vs. Wisc RP: 1.00 |
| Iowa Native Prairie | 3200 | 468.18 | 26.81 | **vs. Kanas NP: 0.03** | 50.08 | 3.47 | **vs. Kanas NP: 0.03** | 3.82 | 0.48 | vs. Kanas NP: 0.70 |
|  |  |  |  | vs. Wisc SG: 0.06 |  |  | **vs. Wisc SG: 0.03** |  |  | vs. Wisc SG: 1.00 |
|  |  |  |  | vs. Wisc RP: 0.14 |  |  | **vs. Wisc RP: 0.03** |  |  | vs. Wisc RP: 1.00 |
| Kanas Native Prairie | 3200 | 570.28 | 56.69 | vs. Wisc SG: 1.00 | 61.62 | 1.17 | vs. Wisc SG: 1.00 | 4.42 | 0.28 | vs. Wisc SG: 1.00 |
|  |  |  |  | vs. Wisc NP: 1.00 |  |  | vs. Wisc NP: 0.14 |  |  | vs. Wisc NP: 1.00 |
| Wisconsin Native Prairie | 3200 | 505.89 | 75.84 | vs. Iowa NP: 1.00 | 56.47 | 3.36 | vs. Iowa NP: 0.08 | 4.25 | 0.37 | vs. Iowa NP: 1.00 |
|  |  |  |  | vs. Wisc SG: 1.00 |  |  | vs: Wisc SG: 0.06 |  |  | vs: Wisc SG: 1.00 |
| Wisconsin Restored Prairie | 3200 | 547.24 | 46.81 | vs. Wisc NP: 1.00 | 61.68 | 1.35 | vs. Wisc NP: 0.11 | 4.18 | 0.23 | vs. Wisc NP: 1.00 |
|  |  |  |  | vs. Kansas NP: 1.00 |  |  | vs. Kansas NP: 1.00 |  |  | vs. Kansas NP: 1.00 |
| Wisconsin Switchgrass | 3200 | 566.33 | 60.33 | vs. Wisc RP: 1.00 | 63.28 | 2.04 | vs. Wisc RP: 1.00 | 4.18 | 0.40 | vs. Wisc RP: 1.00 |

**Supplemental Table 5:** Lipid abundance by microbial group. Data are measured in nmol/g. NP—native prairie, CC—cultivated corn, RP—restored prairie, SG—switchgrass, SE—standard error.

|  | **Arbuscular mycorrhizal fungi** | | **Saprophytic fungi** | | **Gram-positive bacteria** | | **Gram-negative bacteria** | | **Actinomycetes** | |
| --- | --- | --- | --- | --- | --- | --- | --- | --- | --- | --- |
| **Sample** | **Mean** | **SE** | **Mean** | **SE** | **Mean** | **SE** | **Mean** | **SE** | **Mean** | **SE** |
| I-NP | 30.86 | 3.78 | 67.72 | 5.98 | 50.79 | 2.81 | 42.69 | 2.90 | 11.90 | 0.78 |
| I-CC | 4.30 | 0.45 | 10.72 | 1.10 | 26.32 | 1.80 | 20.61 | 1.80 | 9.42 | 1.40 |
| K-NP | 96.35 | 14.23 | 38.26 | 7.70 | 22.71 | 3.70 | 31.84 | 4.25 | 6.58 | 1.33 |
| K-CC | 28.36 | 4.10 | 42.06 | 14.33 | 13.40 | 2.02 | 41.65 | 7.07 | 4.57 | 0.71 |
| W-NP | 50.10 | 6.10 | 58.30 | 10.81 | 37.48 | 5.38 | 51.55 | 7.27 | 12.78 | 1.20 |
| W-CC | 12.60 | 1.17 | 14.98 | 1.41 | 29.13 | 2.31 | 28.07 | 2.33 | 9.20 | 1.03 |
| W-RP | 66.23 | 11.17 | 24.27 | 3.9 | 29.16 | 3.34 | 76.92 | 13.96 | 9.33 | 1.28 |
| W-SG | 35.08 | 5.27 | 13.78 | 1.28 | 28.85 | 2.09 | 42.82 | 5.77 | 10.47 | 1.11 |

**Supplemental Table 6**: Average lipid measurements by cultivation practice (** p-value <0.01, * p-value <0.05)

| **Lipid** | **Cultivated corn (ug/g)** | **Native Prairie (ug/g)** | |
| --- | --- | --- | --- |
| 15:0 ANTEISO | 1.320 | 2.7394 | ** |
| 17:0 CYCLO | 0.629 | 0.7209 |  |
| 17:1 w8c | 0.152 | 1.2426 | ** |
| 18:1 w5c | 0.000 | 0.6314 | ** |
| 18:1 w9c | 5.241 | 12.6702 | ** |
| 12:0 | 0.362 | 0.6267 | ** |
| 12:1 w5c | 0.000 | 0.1043 | ** |
| 13:0 ISO | 0.000 | 0.1874 | ** |
| 14:0 | 0.568 | 0.9186 | ** |
| 14:0 ISO | 0.217 | 0.5418 | ** |
| 14:0 ISO 3OH | 0.051 | 0.0943 |  |
| 14:1 w5c | 0.000 | 0.2884 | ** |
| 15:0 | 0.309 | 0.5959 | ** |
| 15:0 ISO | 2.139 | 2.6496 | * |
| 15:1 ISO G | 0.137 | 0.2987 | ** |
| 16:0 | 8.715 | 14.3186 | ** |
| 16:0 2OH | 0.345 | 0.7877 | ** |
| 16:0 ISO | 0.859 | 1.6120 | ** |
| 16:1 2OH | 0.381 | 0.6763 | * |
| 16:1 Cis Alcohol w7 | 0.038 | 0.0145 |  |
| 16:1 ISO G | 0.209 | 0.2057 |  |
| 16:1 ISO H | 0.000 | 0.5144 | ** |
| 16:1 w5c | 4.151 | 16.5584 | ** |
| 16:1 w7c | 3.369 | 3.2900 |  |
| 16:1 w9c | 0.250 | 0.4713 | ** |
| 17:0 | 0.213 | 0.3369 | * |
| 17:0 ANTEISO | 0.574 | 1.0988 | ** |
| 17:0 ISO | 0.760 | 1.0070 | * |
| 17:0 ISO 3OH | 0.148 | 0.2074 |  |
| 17:1 w6c | 0.000 | 0.3119 | * |
| 18:0 | 1.974 | 2.1820 |  |
| 18:0 2OH | 0.075 | 0.0625 |  |
| 18:1 w9c Alcohol | 0.070 | 0.0784 |  |
| 18:1 w9t Alc(T:17:010M) | 0.239 | 0.3405 |  |
| 18:3 w6c | 0.246 | 0.9777 | ** |
| 19:0 | 0.151 | 0.2201 | ** |
| 19:0 CYCLO 11-12 2OH | 0.000 | 0.1483 | ** |
| 19:0 Cyclo DMA C9-10 | 0.027 | 0.1763 | ** |
| 19:0 ISO | 0.089 | 0.1834 |  |
| 19:0cy c11-12(T:cy | 1.102 | 2.0938 | ** |
| 19:1 w11c | 0.000 | 0.1521 | * |
| 20:0 | 0.945 | 2.3538 | ** |
| 20:1 w9c | 0.071 | 0.3091 | ** |
| 20:4 w691215c | 0.054 | 0.3930 | ** |
| C16:0 N Alcohol | 0.081 | 0.7553 | ** |
| C18 N Alcohol | 0.124 | 0.3551 | ** |
| C20 N Alcohol | 0.438 | 1.3597 | ** |
| i17:1 G(T:16:0 10Me) | 1.779 | 2.1949 |  |
| T:11 methyl 18:1 w7c | 0.182 | 0.2830 |  |
| Unknown 25_052 | 0.024 | 0.3027 | ** |
| Total ug/g | 45.139 | 92.1293 | ** |
| Gram + | 5.869 | 9.6486 | ** |
| Gram - | 8.488 | 11.5622 | * |
| Gm+/Gm- ratio | 0.812 | 0.8416 |  |
| AMF | 4.151 | 16.5584 | ** |
| SF | 6.745 | 16.2477 | ** |
| AMF/SF ratio | 0.655 | 1.3784 | * |
| Protozoal | 0.054 | 0.3930 | ** |
| Actinomycetes | 2.211 | 2.9352 | * |

**Supplemental Table 7.** See excel spreadsheet

**Supplemental Table 8.** See excel spreadsheet

**Supplemental Methods**

**Site Description, including history**

*Iowa sites*

The A.C and Lela Morris Prairie Reserve and a long-term cultivation site were selected in south central Iowa. The parent material is loess. The soil at the prairie site maps as Downs/Gara silt loam (Fine-silty, mixed, superactive, mesic Mollic Hapludalfs) which is very similar to Killduff silt loam (Fine-silty, mixed, superactive, mesic Dystric Eutrudepts) mapped at the cultivated site. However, the onsite inspection classified the sampled site as Tama silt loam (Fine-silty, mixed, superactive, mesic Typic Argiudolls), which is the classification of the immediately adjacent areas (1-2m). The similarity of the sites is further indicated by the USDA National Cooperative Survey listing of Downs (prairie site) and Tama as a “competing” classification for the Killduff soil.

The native prairie site was never cultivated but was grazed. Beginning in 1978 the Jasper County Conservation Board began managing the prairie. No grazing occurred after this date and controlled burns were undertaken every year for the first 3 years, which controlled the encroaching woody vegetation, and reinvigorated the prairie to that historically typical of the region. Burning was undertaken about every second year until the soil was sampled in June of 2009. The last (complete) burn prior to sampling was in spring of 2008. The vegetation at sampling and typical of the last 25 years was 70% big bluestem (*Andropogon gerardii* Vitman) and 30% scattered forbs, which included partridge pea, grey headed coneflower, flowering spurge, black-eyed susan, prairie bush clover, Canada anemone, saw-toothed sunflower, compass plant, bee balm, yarrow, pale purple coneflower, lead plant and prairie blazing star.

The long-term cultivation site is about 10 km from the prairie site and has likely been tilled for more than 100 years until no-till began 4 years before sampling. This site was originally prairie and has been farmed at least since 1882. There are no records of land use from 1880 to 1950, but given the proximity to the homestead, the landscape, fence rows and land use practice of the founding owners, it is likely that this land was under row cropping with crop rotation over much of this period. Since 1950 the land was managed under a soil conservation plan, i.e. farmed on the contour using strip cropping, and was in continuous crop rotation with a rotation of corn-corn-oats-hay-hay. In about 1970 soybeans were added after corn in the rotation and the amount of oats and hay decreased. The moldboard plow was used for row crop seedbed preparation from the 1880’s until about 1975 when minimum tillage (disc) was introduced.

Dairy cow manure was applied annually to the land in the spring since 1950, and probably before, until 1980 when the operation shifted to pig production. Pig manure slurry was injected into the soil until about 2000 when the pig operation was closed. N, P and K fertilizers were regularly applied according to soil test recommendations, and pesticides were used as needed. Genetic modified (GM) corn (*Zea mays*) and soybeans (*Glycine max*) were grown since 2005 and glyphosate herbicide was used on the soybean crop. This site was under no-till soybeans in 2005, no-till corn in 2006, minimum till corn (disc) in 2007, no-till soybeans in 2008 and no-till corn in 2009. Beef cattle manure would have been added after crop harvest for the last 10 years prior to sampling.

*Wisconsin sites*

The native prairie site, known as Goose Pond Prairie, and the cultivated sites are in south central Wisconsin, near Arlington. The soils at the two sites are derived from loess and both are Mollisols. The prairie is on Channahon silt loam (Loamy, mixed, superactive, mesic Lithic Argiudolls) while the cultivated plots are Plano silt loam (Fine-silty, mixed, superactive, mesic Typic Argiudolls).

The native prairie is an approximately 3000-year-old prairie sanctuary owned and managed by the Madison Audubon Society. The sampled area is a remnant prairie that has never been cultivated, and its landscape position just below the crest of a relatively steep slope means it likely did not receive exogenous manure. The remnant site may have been grazed by livestock in the early-mid 20^th^ Century, but the presence of native prairie taxa indicates that if grazing occurred at the site, it likely was not excessive. The site is burned approximately every third year. The dominant vegetation at sampling was sideoats grama, flowery spurge, big bluestem, needle grass, heath aster and Indian grass.

The cultivated sites are about 2 km from the prairie site and located in the Wisconsin Integrated Cropping Systems Trial (WICST) at the Arlington Agricultural Research Station of the University of Wisconsin. Three plant treatments were sampled: 20-year continuous corn, 3-year old switchgrass (*Panicum virgatum*) and 10-year old restored prairie. The 20-year-old continuous corn plot was in a corn-alfalfa rotation (with manure) for decades prior to the mid-1980s.  It was in alfalfa in 1986 and 1987 and in corn in 1988, receiving manure before the alfalfa seeding phase in 1986 and before the corn phase in 1988. The plots were chisel plowed before each new crop.  Also, the land was managed conventionally, i.e. received N fertilizer, herbicides, and insecticides according to standard practice. Lime was added in 1991, but not since. The primary (rough) tillage for continuous corn has been done with a chisel plow (1x) in the fall after corn harvest.  Then the following spring, the secondary (smooth) tillage was done with the field cultivator 1-2x before seeding the new crop. Occasional row cultivation was done if the soil herbicide was ineffective.

The high diversity, restored prairie plot was in a grass waterway (mostly smooth bromegrass (*Bromus inermis*) and quackgrass (*Elytrigia repens*) from 1989 to 1997, prior to restoration. This area was unmanaged other than receiving some mowing to control weeds. In the fall of 1997, it was chisel plowed in preparation for soybean planting in the spring of 1998. That soybean crop was grown with herbicide inputs. After the soybeans were harvested, the area was sprayed with glyphosate and chisel plowed again in the fall of the same year. In the spring of 1999, approximately 2 weeks before planting native prairie, the land was tilled and more herbicide was applied. Finally, a few days before planting, the seedbed was prepared with a field cultivator. After seeding, the soil was firmed up by running a cultipacker over the top. Starting in 1999 and for about 3 years after, the prairie was managed with mowing to control annual weeds. About 26 species were sown at the time, and about 26 species remained at sampling in 2009. While 6 or 7 of the native taxa have been replaced by nonnative colonizers, e.g. smooth brome (*Bromus inermis*), Kentucky bluegrass (*Poa pratensis*), native prairie species remain the dominant taxa.

The current switchgrass plots share the same history with the prairie plots until 1999. They were in corn (harvested as grain) from 1999-2006 and managed as a conventional (annual tillage), high-input crop. In the spring of 2007, the old corn plots were sprayed and switchgrass was no-till drilled in August 2007. Some herbicide was applied in early May of 2008 and weeds were chopped in mid-June. The switchgrass established well and remains the dominant taxon.

*Kansas sites*

The native prairie site was located north of Manhattan, KS (39^o^ 12’N, 96^o^35’W, 293 m above M.S.L). Vegetation on the site was a mixture of C_3_ and C_4_ species, dominated by big bluestem (*Andropogon gerardii* Vitman) and indiangrass (*Sorghastrum nutans* (L.) Nash). Members of the sedge family made up to 10% of the composition. Principal forbs included ironweed (*Vernonia baldwinii* var. *interior* (Small) Schub.), western ragweed (*Ambrosia psilostachya* DC.), Louisiana sagewort (*Artemisia ludoviciana* Nutt.), and manyflower scurfpea (*Psoralea tenuiflora* var. *floribunda* (Nutt.) Rydb.). Average aboveground peak biomass of 425 g m^-2^ occurs in early August, of which 35 g m^-2^ is from forbs (Owensby and Anderson, 1967). Soils in the area are transitional from Ustolls to Udolls (Tully fine, mixed, superactive, mesic Pachic Argiustolls). Slope on the area is 5%. Fire has been infrequent, occurring 2-3 times in 10 yr. Past history has included primarily winter grazing by cow-calf pairs.

The no-till soil was sampled from a long-term tillage N source study in continuous corn (*Zea mays*) established in 1990 at the North Agronomy Farm located at Kansas State University (39º12'N, 96º35' W, 296 m M.S.L.), Manhattan, KS. The soil was a moderately well-drained Kennebec silt loam (Fine silty, mixed mesic Cumulic Hapludolls) previously cropped to oats (Avena sativa). Treatments were arranged in a completely randomized split plot design with four replications. Tillage treatments included NT and chisel-disk (CT; fall chisel plow and spring offset disk). Applying 321 g L^-1^ of atrazine (2-chloro-4-ethylamine-6-isopropylamino-S-triazine) and 400 g L^-1^ of metolachlor [2-chloro-6’-ethyl-N-(2-methoxy-1-methylethyl) acet-o-toluidide] (Bicep 6L, Ciba-Geigy) controlled weeds at the rate of 4.76 L ha^-1^ within one month of corn emergence. Nitrogen fertilizer was applied as urea at 168 kg N ha^-1^ yr^-1^.

**Metagenome Assembly**

*Iowa native prairie*

327 Gbp of Illumina data was converted to a format suitable for the Convey graph constructor running on A Convey HC-1 using ‘cnygc -unifySeq -trimB -shortPaired’ resulting in a CnyUnifiedSeq file of 258808269534 bp of trimmed data with an average length of 69bp. A velvet-compatible roadmap file was constructed with ‘cnygc -threads 4 -logStats -roadmap -partition 1of32’ which creates 32 partitions of the initial CnyUnifiedSeq file. The roadmap was converted to a graph using ‘cnygc -graph -logStats -threads 32 -partitionCnt 32’. Finally the resulting graph was converted to 10744470 contigs comprising 3025478413 bp with ‘velvetg . -cov_cutoff 4 -ins_length 300 -exp_cov auto’

*Iowa continuous corn*

219 Gbp of Illumina data was produced for this sample but all of library HTHO failed QC leaving 197Gbp of Illumina GA2 (2x114bp, 2x76bp) and GAIIx (2x100bp) sequence for assembly. Data was converted on the HC-1 using ‘cnygc -trimB -shortPaired -fastq.gz’ to CnyUnifiedSeq format having an average length of 74bp with 2.055 billion reads surviving. A 32-way partitioned roadmap file was produced using ‘cnygc -roadmap -partition’ with K=61 on the HC-1. The resulting roadmap files were converted to an intermediate graph on an IBM X3850 with 1 TB of RAM and finally to contigs using ‘velvet -cov_cutoff 4 -ins_length 300 ‘ (version 1.2.03). The resulting assembly contained 2716529 contigs covering 918 Mbp.

*Kansas native prairie*

A total of 32 lanes of Illumina GA2 (2x76bp, 2x114bp), GAIIx (2x100bp, 2x114, 2x150bp) and HiSeq-2000 v2 or v3 (2x100bp, 2x150bp) produced 598Gbp in 5099547628 reads. After removing low quality regions using ‘cnygc -trimB’, and K=51, the average length was 95bp. A 32-way partitioned roadmap was created using ‘cnygc -threads 4 -roadmap -partition 32’ on the HC-1. The resulting roamap files were converted to an intermediate velvet-compatible graph with ‘cnygc -graph’ on an IBM X3850 with 1TB of RAM. The graph was converted to contigs with ‘velvetg -cov_cutoff 4 -ins_length 300 -exp_cov auto’ (version 1.2.03) and produced 2778673 contigs covering 92832547 bp.

*Kansas continuous corn*

Sample was sequenced in using GA2 (2x114) GAIIx (2x100). A total of 30 lanes of sequence was produced containing 271.111Gbp and 2677222281 reads. After trimming regions of reads containing only quality value ‘B’ using ‘-trimB’, the average length was 78. Reads were pre-processed and a CnyUnifiedSeq file was created using ‘cnygc -unifySeq -trimB’, then 32 roadmap files were created from this file using ‘cnygc -roadmap -partition 32’ using K=61. Roadmap files were converted to an intermediate graph with ‘cnygc -graph -threads 32’ on an IBM X3850 with 1GB or RAM. 5020643 contigs covering 13420462 bp were produced with ‘velvetg. -cov_cutoff 2 -ins_length 300 -exp_cov auto’ with velvet version 1.2.03 on an IBM X3850 with 1TB of RAM.

*Wisconsin native prairie*

Sequenced on Illumina GA2 (2x114) and GAIIx (2x100), a total of 19 usable lanes produced 199.025Gbp and 2098317886 reads. Prior to assembly, reads were quality filtered and trimmed to 60bp or 80bp depending on the lane quality. Data was converted to CnyUnifiedSeq using ‘cnygc -trimB’, then partitioned into 16 roadmap files using ‘cnygc -partition 16’, and an intermediate graph was produced using ‘cnygc -graph’ with K=61 on the Convey HC-1. Velvetg (version 1.2.03) produced 2013614 contigs covering 579469157bp.

*Wisconsin continuous corn*

One library was sequenced on Illumina GA2 (2x75, 2x114) or GAIIx (2x100) to produce 19 lanes and 192.225Gbp of sequence and 1912865700 reads. After removing low quality regions with option cnygc ‘-trimB’ , the average read length was 83. The resulting CnyUnifiedSeq file was converted to 32 roadmap partitions using ‘cnygc -partition 32’ with K=51 all on the Convey HC-1. An intermediate graph was constructed with ‘cnygc -graph’ on a Dell R910 with 1TB of RAM and produced 3942652 contigs covering 1461086476 bp with ‘velvetg . -cov_cutoff 4 -ins_length 300 -exp_cov auto’ and velvet version 1.2.03.
